# Bubble Nasal Continuous Positive Airway Pressure (bNCPAP): An Effective Low-Cost Intervention for Resource-Constrained Settings

**DOI:** 10.1155/2020/8871980

**Published:** 2020-09-08

**Authors:** Walufu Ivan Egesa, William Mugowa Waibi

**Affiliations:** Department of Paediatrics and Child Health, Faculty of Clinical Medicine and Dentistry, Kampala International University, P.O. Box 20000 Kampala, Uganda

## Abstract

Preterm birth complications are responsible for almost one-third of the global neonatal mortality burden, and respiratory distress syndrome remains the single most common cause of these preventable deaths. Since its inception, almost half a century ago, nasal continuous positive airway pressure (NCPAP) has evolved to become the primary modality for neonatal respiratory care in both the developed and developing world. Although evidence has demonstrated the effectiveness of low-cost bubble NCPAP in reducing newborn mortality, its widespread use is yet to be seen in resource-constrained settings. Moreover, many tertiary hospitals in developing countries still utilise an inexpensive locally assembled bNCPAP system of unknown efficacy and safety. This review provides a brief overview of the history, physiological benefits, indications, contraindications, and complications of bNCPAP. Evidence regarding the effectiveness of low-cost bNCPAP in the neonatal intensive care unit is also summarised. The article further details a locally assembled bNCPAP system used in resource-constrained settings and highlights the care package for neonates receiving bNCPAP, failure criteria, and strategies for weaning.

## 1. Introduction

More than 60 percent of the 15 million preterm births in 2010 occurred in sub-Saharan Africa and Asia [1]. Complications attributed to preterm birth are globally recognised as the leading cause of neonatal mortality [2]. In 2018, it was estimated that preterm birth complications were responsible for 18% of under-five deaths and 35% of the 2.5 million neonatal deaths worldwide [3]. The leading cause of these deaths is respiratory distress syndrome (RDS), a result of pulmonary surfactant deficiency due to lung immaturity [4, 5]. Scientific advancements such as the use of antenatal corticosteroids, exogenous surfactant therapy, continuous positive airway pressure (CPAP), and mechanical ventilation have resulted in significantly improved survival of neonates with respiratory distress in high-income countries [6, 7]. On the other hand, mechanical ventilation is invasive and has been linked to several short- and long-term morbidities such as bronchopulmonary dysplasia (BPD) [8]. Moreover, conventional CPAP devices and mechanical ventilation are expensive options, and mechanical ventilation requires high-level expertise and trained personnel, which is not currently possible in many resource-limited countries [9]. Although several conventional bubble nasal CPAP (bNCPAP) devices are available, they may cost up to US$6,000 to US$10,000 [10, 11], while a low-cost bNCPAP system may cost as low as US$350 to US$2000 [12, 13]. Comparatively, bNCPAP may cost approximately 15% of the cost of the cheapest mechanical ventilator [11].

To achieve the Sustainable Development Goals (SDGs) for child survival, developing countries need to address these preventable newborn deaths by scaling up efforts to implement innovative and yet effective low-tech interventions [2, 3]. The widespread use of oxygen therapy and simple inexpensive systems that provide bNCPAP for neonates with respiratory distress may significantly lower neonatal mortality in developing countries [2, 6].

## 2. History of CPAP

Almost 50 years ago, Gregory and associates revolutionised neonatal care with the introduction of the first CPAP system intended for neonatal use [14]. During the early years of its introduction, CPAP was administered using an endotracheal tube, sealed head chamber (Gregory box), or face mask [14]. It was not until 1973 that the use of nasal prongs was described [15]. Since then, nasal prongs have remained the most predominantly used interface [16]. Mechanical ventilators designed for neonatal use were introduced during the late 1970s, replacing CPAP as the initial respiratory support strategy for premature infants with lung disease for nearly four decades [17, 18]. However, the advent of the twenty-first century came with a reemerging interest in gentler noninvasive ventilation modalities such as CPAP. This followed an in-depth understanding that mechanical ventilation plays a role in the pathogenesis of BPD in premature infants [18, 19].

## 3. Physiologic Benefits of NCPAP

As the name implies, NCPAP functions by maintaining a consistent inspiratory and expiratory pressure above the ambient pressure for spontaneously breathing neonates. This results in a reduction in upper airway resistance and collapsibility, increased diaphragmatic tone and contractility, improved alveolar recruitment and lung compliance, increase in functional residual capacity (FRC), improved ventilation/perfusion, and reduction in oxygen requirement. NCPAP also splints the airways and diaphragm, stabilizing the chest wall, and consequently slows and regularises the respiratory rate. Furthermore, NCPAP improves the production and conservation of surfactant on the alveolar surface, reduces alveolar oedema, and improves lung growth [20, 21].

## 4. Indications and Contraindications for NCPAP

Bubble CPAP is indicated for use in neonates with respiratory distress syndrome, transient tachypnea of the newborn, meconium aspiration syndrome, congenital pneumonia, respiratory distress due to perinatal asphyxia, and postextubation following mechanical ventilation [22–26]. It is also effective in neonates with congenital or acquired airway lesions that are prone to collapse, such as tracheomalacia [18]. The clinical indications and contraindications [8, 18, 27–29] for NCPAP use among preterm and term neonates are shown in Table 1.

## 5. Effectiveness of Low-Cost bNCPAP

Bubble NCPAP is an effective noninvasive ventilation strategy for newborns with mild-to-severe respiratory distress and needs to be scaled up in resource-constrained countries [10, 26, 30–32]. It can be successfully introduced and independently operated by neonatal intensive care unit (NICU) nurses after a relatively short period of training [11, 31, 33]. The effectiveness of various bNCPAP devices in reducing neonatal respiratory failure, need for surfactant, mechanical ventilation, and mortality has been evaluated by heterogeneous studies in both developed and developing countries [11, 32, 34–36]. In fact, low-cost bNCPAP devices may be comparable in performance to standard bNCPAP systems used in the developed world [13, 36–38], although some relatively inexpensive bNCPAP devices designed for resource-limited settings are of high resistance, low pressure stability, and impose increased work of breathing [39].

In Blantyre, Malawi, a prospective, nonrandomized controlled study found a 71% survival rate among neonates with severe respiratory distress who received low-cost bNCPAP, compared to 44% among those who received standard nasal oxygen therapy. Overall, two-thirds (65.5%) of very low birth weight (VLBW) neonates who received bNCPAP therapy survived to discharge, compared to 15.4% among the controls. A significant beneficial effect of bNCPAP was also observed among neonates with RDS, and sepsis [40]. A prospective pre- and postintervention study to determine the impact of low-cost bNCPAP in Nicaragua demonstrated a significant reduction in mortality (40 vs. 23%; *p* < 0.0001) and rates of mechanical ventilation (72 vs. 39%; *p* < 0.0001). However, an increase in the mean duration of NICU stay was observed (14.6 days in 2006 vs. 17.5 days in 2008), as was the proportion of neonates who were exclusively treated with bNCPAP (27% vs. 61%) [41]. Similarly, the introduction of low-cost bNCPAP in a tertiary hospital NICU in Eastern Uganda resulted in a 44% reduction in VLBW infant mortality associated with RDS (OR 0.56, 95% CI 0.36–0.86; *p* = 0.01) [42]. A recent randomised controlled trial (RCT) involving preterm neonates at a tertiary hospital in Northern Tanzania evaluated the efficacy and treatment outcomes of a low-cost bNCPAP system compared to oxygen therapy. In this study, neonates were started on bNCPAP if the Silverman-Andersen Respiratory Severity Score was ≥6, whereas a score of ≤3 for at least 6 hours was considered for weaning off bNCPAP. Neonates who received bNCPAP had higher survival compared to those who received oxygen therapy (77.3% vs. 47.8%), although this difference was not statistically significant [43].

## 6. Complications of NCPAP

Facial, nasal bridge and septal injury are the most common complications, occurring more frequently among preterm infants who require NCPAP for a prolonged duration [36, 37, 43–45]. Based on severity, nasal injury is classified into three stages: nonblanching erythema on an otherwise intact skin, superficial erosion, and full thickness necrosis of the skin [46, 47]. The reported incidence of nasal injury associated with NCPAP ranges from 6.4% to 91.6% [36, 46, 48] and is inversely related to the gestational age and birth weight [46]. Numerous RCTs and meta-analyses have demonstrated an association between the type of interface and nasal injury during noninvasive ventilation [48–51]. Short binasal prongs and nasal masks are the most widely used interfaces, but nasopharyngeal tube and nasal cannula have also been utilised [29, 52–54]. The merits and demerits of these interfaces are shown in Table 2. Nasal mask is generally associated with a significantly lower risk of nasal injury compared to binasal prongs [48–51]. Nasal masks apply excessive constant pressure to the nasal bridge and philtrum, reducing local tissue perfusion and causing nasal injury [50]. On the contrary, binasal prong injury is mainly observed at the columella and anterior part of nasal septum [18, 50, 55], which may be due to constant pressure between the two prongs [50]. Whilst long-term sequelae are uncommon, nasal injury may cause permanent disfigurement and functional sequelae such as nasal vestibular stenosis, nasal deformity, and visible scars requiring cosmetic surgery [46, 56].

Irrespective of the device used, leaks at the nares-prong interface are common and need to be minimized if consistent CPAP is to be delivered [57]. The occurrence of nasal injury and air leak can be reduced by using nasal masks [50], nasal prongs that are of sufficient size to snugly fit in the nostrils without causing blanching [25], and application of hydrocolloid dressing [58], although the latter has not been extensively studied.

Other NCPAP-associated complications include nasal irritation [31], dislodgement of nasal prongs, mucous obstruction of the airway, overdistended lungs, pneumothorax [9, 59], and abdominal distension, also referred to as “CPAP belly syndrome” [45, 60]. Studies to evaluate the hemodynamic alterations of NCPAP have reported changes such as impediment of systemic and pulmonary venous return to the heart, although NCPAP does not affect left ventricular output, pulse rate, and mean arterial pressure [61].

## 7. How to Set Up an Improvised bNCPAP System

The simplest bNCPAP setup is composed of an air compressor, air-oxygen blender, humidifier chamber, and tubing with patient interface [11, 29, 62]. Initially, a fraction of inspired oxygen (FiO_2_) of 0.30 for neonates <28 weeks gestation, 0.21–0.30 for 28–31 weeks, and 0.21 for ≥32 weeks gestation is used [63]. If oxygenation is still compromised, the FiO_2_ is increased by steps of 0.05 to a maximum of 0.8 [29]. Positive end-expiratory pressure (PEEP) is usually started at 4-6 cm H_2_O and can be adjusted depending on the neonate's clinical condition, oxygenation, and perfusion [29, 63]. If there is no improvement, PEEP is increased by 1-2 cm H_2_O up to a maximum of 7-8 cm H_2_O [29].

**Figure 1:**
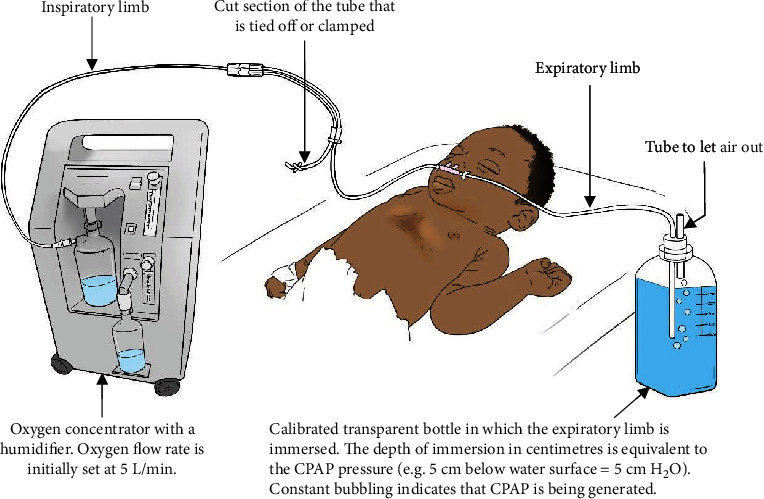
Illustration of a low-cost improvised bNCPAP circuit.

However, this article describes a method of locally assembled bNCPAP that is used in resource-constrained settings (Figure 1), as described by studies in Nigeria, India, and Pakistan. The average cost of this system lies between US$3.00 and US$12 [64–66].

The bNCPAP is set up using an oxygen concentrator or oxygen cylinder, and modified low resistance binasal prongs [12, 67, 68]. The tube is cut on one side of the prong, and the free end serves as the expiratory limb, while the short end of the cut section is firmly tied off and glued or clamped to avoid air leak. The second tubing on the other side of the prong serves as the inspiratory limb [12, 66]. There are however two major setbacks for locally made circuits. They lack an air-oxygen blender, and thus, titrating oxygen is not possible. However, this may be solved using an oxygen concentrator with an air-oxygen blender [67]. Secondly, the oxygen gas flow rate needed to generate CPAP, usually 5–10 L/min, may be too high for the diameter of regular nasal prongs, generating too much resistance and failing to produce an appropriate level of pressure [10].

### 7.1. Oxygen Sources

Oxygen (O_2_) concentrators and cylinders are the two common sources of O_2_ for bNCPAP. Oxygen cylinders contain liquid O_2_ that is distilled at very low temperatures and high pressures. The oxygen flow rate is usually started at 5 L/min and may be varied as needed up to 10 L/min, while looking for bubbles in the calibrated transparent container [68]. Unfortunately, using high-concentration oxygen can cause retinopathy of prematurity and is thus not safe for preterm neonates born before 32 weeks of gestation [67]. The use of 100% concentration of oxygen should be avoided in all neonates, unless a blender is available. A pulse oximeter can guide the titration of the FiO_2_, because hyperoxygenation can lead to free radical damage [63].

### 7.2. Inspiratory Limb

The inspiratory limb is the section of the breathing circuit that is connected to the humidified oxygen source and the patient interface [10, 64].

### 7.3. Expiratory Limb

The expiratory limb for bNCPAP is a tube of noncollapsible plastic leading from the patient interface to the pressure generator where it is immersed in water [68]. In this case, the free section of the tube is immersed (through a straight straw) into a calibrated transparent container (or saline bottle) containing distilled water and the depth of immersion is equivalent to the CPAP pressure in cm H_2_O, usually set at 5 cm H_2_O [10, 66, 68]. In the event that the expiratory circuit is short, a section of an intravenous fluid administering tubing is cut and attached, then immersed in water [69]. The tube is then secured to the bottle with an adhesive tape to ensure that the immersion length remains constant, also ensuring that it does not touch the bottom of the bottle [64, 66]. Before the system is connected to the neonate, the oxygen flow meter is set at 5-8 L/min, and the nasal prongs are blocked to test for bubbling [66]. The presence of constant bubbling indicates that positive airway expiratory pressure is being generated [10, 66, 68]. An opening or tube on the bottle allows air to escape [67].

### 7.4. Patient Interface

Short binasal prongs of appropriate size are used to set up the CPAP circuit [69]. This is because small prongs do not provide the much-needed seal to generate the desired CPAP. To prevent air leaks, the patient interface (prongs) are firmly applied to the nostrils by gentle placement of strapping [64].

## 8. Care of Infants on NCPAP

Besides ensuring that the equipment is appropriately fitted, neonatal nurses and doctors need to carefully monitor infants receiving bNCPAP by performing continuous assessment of the infant's clinical state and response to therapeutic measures [43, 59, 70]. Serial evaluation of the infant's respiratory, cardiovascular, and neurological status helps detect any serious changes that require immediate intervention, thus minimising bNCPAP failure and complications [43, 71]. The Clinical Respiratory Distress Scoring System (Downes score) and Silverman-Andersen Respiratory Severity Score are the two commonly used tools for grading the severity of respiratory distress and for monitoring clinical improvement [31, 72–74]. The Downes score (Table 3) reliably correlates with arterial blood gases (ABGs) and is thus a very useful adjunct in settings where ABGs are not available [75]. Using a pulse oximeter, the nursing team should monitor the heart rate; oxygen saturation (SpO_2_), preferably preductal; respiratory rate; and temperature every 2 to 3 hours, unless the clinical state of the neonate requires more frequent monitoring [71]. According to the European Consensus Guidelines on the management of RDS, oxygen saturation should be maintained at 90-94%, with a set alarm limit of 89-95%. The core body temperature range should be maintained between 36.5°C and 37.5°C [63], and the neonate's blood pressure, capillary refill time, and urine output need to be monitored [29].

The neonate's head should be elevated by 30° and airway patency maintained by gently suctioning the nostrils, mouth, pharynx, and nasal prongs every 4 hours or as needed to clear the mucous [29, 43, 71]. To prevent hypothermia and damage to the nasal mucosa, the humidifier temperature should be set at 37°C. After every 2 to 4 hours, infants on NCPAP should be repositioned, to maintain the integrity of skin. Furthermore, increment in abdominal girth should be monitored, and an orogastric tube passed for gastric decompression [33, 43, 71]. Feeding should be done cautiously, and when ideal, infants may be allowed to breastfeed [71, 76]. After feeding, the proximal end of the orogastric tube should be closed for 30 minutes and then kept open for the next 90 minutes, if the infant is fed every 2 hours [29].

Because of the risk of nasal injury and air leak, nurses need to provide meticulous care of the skin and nasal septum, through regular monitoring of the nasal skin, proper anatomical placement of the prongs inside the nostrils, ensuring a distance of 2 mm between the nasal septum and the prongs, delivering humidified gas, using a tape to secure the nasal prongs, daily gentle massage for the nasal septum and bridge, use of hydrogel to lubricate the nasal skin, and antimicrobial ointment for skin breakdown [25, 34, 44, 70, 77]. These prevention measures for nasal breakdown are more likely to be successful if standardised protocols are implemented [77].

## 9. Failure of bNCPAP

Bubble NCPAP failure has been linked to a high risk of mortality and morbidity, and yet, the rates of NCPAP failure are significantly high among extremely preterm neonates (<28 weeks gestation), a group that carries the highest risk of BPD [78, 79]. Failure of NCPAP is defined as one or more of the features shown in Table 4. Early identification of the predictors of NCPAP failure is crucial for targeted escalation of interventions to surfactant therapy and mechanical ventilation [79]. For preterm neonates being managed for RDS, NCPAP failure requiring mechanical ventilation is more likely to occur if antenatal corticosteroids were partially given or not administered at all, radiographic evidence of severe RDS, patent ductus arteriosus, sepsis/pneumonia, FiO_2_ ≥ 0.5, or a Downes score > 7 after 15 to 20 minutes of NCPAP [80, 81], and if nasal mask is not used as the interface [51].

## 10. Weaning Off from NCPAP

### 10.1. Timing of Weaning

Optimal timing of weaning from NCPAP is important. Early weaning expedites the transition from intensive care to special care, may reduce treatment cost [82], and prevent CPAP-associated complications. Therefore, a decision to wean a neonate off CPAP should be based on the presence of predefined stability criteria (Table 5), which should all be present for 24–48 hours prior to weaning [83]. In a retrospective study of 454 preterm neonates with a mean gestational age of 29.3 ± 2.2 weeks and mean birth weight of 1357 ± 392 grams, Rastogi and associates established that NCPAP can be successfully weaned off at a mean postmenstrual age (PMA) of 32.9 ± 2.4 weeks and body weight of 1611 ± 432 grams [84]. A meta-analysis of six randomised controlled trials and one retrospective chart review concluded that successful weaning from NCPAP occurs at a PMA of 32 to 33 weeks and weight of 1600 grams [83].

### 10.2. Weaning Strategies

Available weaning options include sudden discontinuation, gradual pressure wean, discontinuing bNCPAP for a predetermined number of hours each day and gradually increasing the amount of time off bNCPAP each day until it is stopped completely (gradually graded time off wean), replacing bNCPAP with high or low flow oxygen, or a combination of these methods [83, 85–87]. Since there are no consensus guidelines regarding the optimum timing and method of weaning, there exist substantial differences in the weaning practices for bNCPAP among health facilities and medical workers [86, 88]. Moreover, studies to determine the optimum strategy for weaning off NCPAP have yielded conflicting results. In Australia, a multicentre RCT found that weaning off from NCPAP with the intention to remain off significantly reduced the weaning time, NCPAP duration, oxygen duration, BPD, and length of hospital stay [89]. Similar results were reported by Jeffery and associates who found that ceasing NCPAP using standard protocol with a goal to remain off rather than slow weaning significantly reduces NCPAP time, PDA, and BPD among preterm neonates born at <30 weeks [82]. In New York (USA), a single-centre RCT involving 56 preterm neonates born at ≤32 weeks found no significant difference in the success of gradual weaning using graded time off NCPAP versus sudden weaning [86]. A similar study of preterm neonates born at ≥28 weeks in Egypt found no difference in the success of NCPAP weaning between sudden weaning and gradual transition using nasal cannula. In this study, preterm infants weaned from NCPAP to nasal cannula were more likely to have increased exposure to oxygen and longer duration of respiratory support compared to those who are weaned to room air [90]. Conversely, an RCT involving neonates born at 26 to 32 weeks gestation in a level III NICU reported a higher success rate of gradual weaning compared to sudden weaning during the first attempt [91]. During weaning, upper airway patency should be maintained through measures such as suctioning and keeping the neck in neutral position [85].

### 10.3. Failure of Weaning

Successful weaning is determined by the absence of persistent tachypnea, marked retractions, and apneic episodes on room air without any respiratory support or supplemental oxygen for 7 days [86]. Proposed clinical criteria for defining failed trial off CPAP are shown in Table 6. Factors such as low birth weight, chorioamnionitis, prior intubation, anemia, apnea, grade 3 and 4 intraventricular hemorrhage, sepsis/necrotising enterocolitis, patent ductus arteriosus, and gastroesophageal reflux have been found to influence the success of weaning from NCPAP [83, 84]. On the other hand, methylxanthines such as caffeine have proven to be useful adjuncts during weaning [88].

## 11. Summary and Conclusion

Respiratory distress syndrome is the most common cause of death among preterm neonates. Low-cost bNCPAP has been proven to be an effective and safe intervention for improving oxygenation of neonates with respiratory distress and abates the need for invasive ventilation, which has been linked to bronchopulmonary dysplasia. This intervention should be considered as a primary modality of respiratory support for neonates in resource-constrained settings. However, many tertiary institutions in developing countries still rely on locally assembled bNCPAP systems whose efficacy and safety have not yet been well studied. To further improve the survival of neonates with respiratory problems, clinicians and NICU nurses need to understand the optimum care package for neonates receiving bNCPAP and measures for prevention of bNCPAP-associated complications. To date, there is no consensus regarding the weaning strategy. Nonetheless, adequately powered randomised trials are needed to investigate the most ideal strategy and timing of weaning from bNCPAP.

## Figures and Tables

**Table 1:** Indications and contraindications for NCPAP.

Indications	Contraindications
Respiratory distress syndrome	Progressive respiratory failure (pH < 7.2, PCO_2_ > 65 mmHg)
Apnea of prematurity (obstructive apnea)	Poor respiratory drive with frequent apnea or bradycardia not improved by CPAP
Transient tachypnea of the newborn	Severe cardiovascular instability (hypotension)
Respiratory distress due to perinatal asphyxia	Congenital malformations (choanal atresia, cleft lip and palate, Pierre Robin sequence, congenital diaphragmatic hernia, and tracheoesophageal fistula)
Meconium aspiration syndrome	
Congenital pneumonia	
Postextubation in preterm VLBW infants	
Laryngomalacia/tracheomalacia/bronchomalacia	

References: [8, 18, 27–29].

**Table 2:** Comparison of CPAP interfaces.

Interface	Advantages	Disadvantages	Remarks
Nasal prongs	Simple device	Easily get dislodged from the nose	Examples include Argyle, Hudson, Infant Flow Driver (IFD), and INCA prongs
	Lower resistance leads to greater transmission of pressure	Risk of trauma to nasal septum and turbinates	
	Mouth leak acts like a “pop-off” mechanism	Variable end expiration pressure due to nares-prong air leaks	
Nasal mask	Minimal risk of nasal trauma	Difficulty in obtaining an adequate seal	
	More effective in preventing intubation and mechanical ventilation		
Nasal cannula	Easy to apply	Unreliable pressure delivery	Cannula with outer diameter of 3 mm and flows up to 2 L/min can be used to treat apnea of prematurity
	Well tolerated	May need high flows to generate pressure if only oxygen is used (without air blender)	
		Delivered FiO_2_ may be high	
		Large air leaks around the cannulae	
Nasopharyngeal tube	Easily available	Easily blocked by secretions	A cut endotracheal tube may be used.
	Economical	Likely to kink	The length is estimated by measuring the distance from the ear lobe to the tip of the chin or nose.
	More secure fixation		Confirm tube placement by visualizing its tip behind the uvula.

References [29, 49, 53, 54].

**Table 3:** Downes score.

Clinical parameter	Score		
	0	1	2
Respiratory rate (per minute)	60	60-80	>80 or apneic episodes
Cyanosis	None	In room air	In 40% inspired oxygen concentration
Retractions	None	Mild	Moderate to severe
Grunting	None	Audible with stethoscope	Audible without stethoscope
Air entry^∗^ (crying)	Clear	Delayed or decreased	Barely audible

The RDS score is the sum of the individual scores for each of the five observations. ^∗^Air entry represents the quality of inspiratory breath sounds as heard in the midaxillary line. FiO_2_: fraction of inspired oxygen. Reference [75].

**Table 4:** Failure criteria for NCPAP.

Requirement of pressure >8 cm H_2_O
FiO_2_ requirement >0.6
PaO_2_ < 50 mmHg on maximum acceptable settings
PaCO_2_ > 65 mmHg and pH < 7.2 on maximum acceptable settings
Air leak on NCPAP
Recurrent apnea on NCPAP despite caffeine citrate or aminophylline.

FiO_2_: fraction of inspired oxygen; PaO_2_: partial pressure of arterial oxygen. Reference [24].

**Table 5:** Stability criteria for weaning from NCPAP.

CPAP pressure of 5 cm H_2_O
FiO_2_ of 0.21 (room air) required to maintain SPO_2_ > 90%
Normal work of breathing: no persistent tachypnea (>60 breaths for >2 h), no significant chest retractions
No apnea (cessation of respiration for >20 seconds) associated with bradycardia or cyanosis with >2 episodes in 12 h or >3 in 24 h with at least one requiring bag and mask ventilation
Saturation > 90% most of the time or PaO_2_/transcutaneous PaO_2_ > 45 mmHg
Not currently treated for PDA or sepsis
Tolerated time off CPAP during nursing care ≥15 min.

PDA: patent ductus arteriosus; FiO_2_: fraction of inspired oxygen; PaO_2_: partial pressure of arterial oxygen; SPO_2_: oxygen saturation. References [82, 91].

**Table 6:** Clinical criteria for defining failed weaning from NCPAP (at least 2 of the following).

Increased work of breathing: persistent tachypnea (>60 for >2 h) and marked retractions
Apnea associated with bradycardia or cyanosis with >2 episodes in 12 h or >3 in 24 h with at least one requiring resuscitation
Increased O_2_ requirement >0.21 to maintain the oxygen saturations >90%
Abnormal blood gases (2 arterial samples taken >2 h apart) with low pH < 7.2, PaCO_2_ > 65 mmHg, PaO_2_ < 50 mmHg

PaCO_2_: partial pressure of arterial carbon dioxide; PaO_2_: partial pressure of arterial oxygen. References [82, 91].
